# A study exploring the causal relationship between glaucoma and anxiety disorders

**DOI:** 10.3389/fmed.2024.1410607

**Published:** 2024-08-07

**Authors:** Bin Lin, Meng Xu, Long-long Chen, Dong-kan Li

**Affiliations:** ^1^Xiamen Eye Center and Eye Institute of Xiamen University, Xiamen, China; ^2^Xiamen Clinical Research Center for Eye Diseases, Xiamen, Fujian, China; ^3^Xiamen Key Laboratory of Ophthalmology, Xiamen, Fujian, China; ^4^Fujian Key Laboratory of Corneal and Ocular Surface Diseases, Xiamen, Fujian, China; ^5^Xiamen Key Laboratory of Corneal and Ocular Surface Diseases, Xiamen, Fujian, China; ^6^Translational Medicine Institute of Xiamen Eye Center of Xiamen University, Xiamen, Fujian, China

**Keywords:** glaucoma, anxiety disorders, causal relationship, Mendelian Randomization, GWAS datasets

## Abstract

**Background:**

Glaucoma, a leading cause of global blindness, is characterized by optic nerve damage and visual field loss. Previous studies have suggested a potential association between glaucoma and anxiety disorders. However, the causal relationship between these two conditions remains unclear.

**Methods:**

In this study, we conducted a Mendelian Randomization analysis to investigate the causal relationship between glaucoma and anxiety disorders. We sourced Genome-Wide Association Study (GWAS) datasets for glaucoma and anxiety with the largest sample sizes from the Integrative Epidemiology Unit OpenGWAS (IEU OpenGWAS) project website. Instrumental variables were selected based on specific criteria, and statistical analyses were performed using the R programming language.

**Results:**

After filtering and merging the datasets, a total of 60 Single Nucleotide Polymorphisms (SNPs) were obtained for analysis. Regression models were applied to assess the causal relationship between glaucoma and anxiety disorders. The results from all four methods indicated that glaucoma does not cause anxiety disorders (*p* > 0.05).

**Conclusion:**

Through rigorous Mendelian Randomization analysis, our findings indicate that glaucoma is not a causative factor for anxiety, with minimal influence from confounding factors in this study. These findings enhance our understanding of the relationship between glaucoma and anxiety.

## 1 Background

Glaucoma, a leading cause of global blindness, is characterized by optic nerve damage and visual field loss, often culminating in blindness (1). With multifactorial etiology, it affects approximately 5.2 million individuals worldwide, constituting 15% of the global burden of blindness (2). Moreover, due to population aging, its prevalence is expected to rise, projected to affect approximately 112 million people by 2040 (3).

Anxiety disorders represent the most prevalent mental health issues globally, significantly impacting individuals’ quality of life, work productivity, and societal well-being. Despite being distinct diagnostic entities, anxiety often coexist clinically, demonstrating high comorbidity (4). Globally, it is estimated that 3.7% of individuals will experience Generalized Anxiety Disorder (GAD) at some point in their lives (5). The impact of GAD on functioning and quality of life is comparable to, or even greater than, the effects associated with severe depression and substance abuse disorders (6).

In recent years, increasing attention has been directed toward the high prevalence of anxiety among individuals with glaucoma. These studies suggest that glaucoma is not solely a visually impairing ocular condition but may also be linked to patients’ psychological well-being, indicating a close interplay between the two (7–10).

**FIGURE 1:**
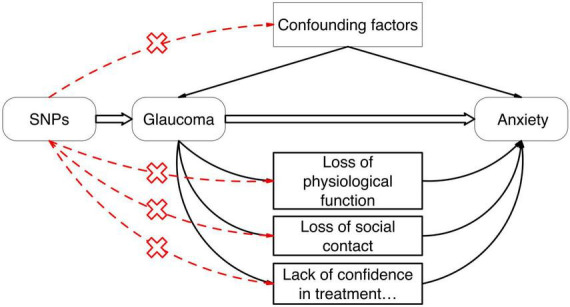
The causal relationship between Glaucoma and anxiety can be further confirmed by Mendelian Randomization studies and the effect of confounding factors can be excluded.

In our research, we employed the two-sample Mendelian Randomization (MR) approach, leveraging Single Nucleotide Polymorphisms (SNPs) as instrumental variables derived from Genome-Wide Association Study (GWAS) summary statistics. This methodology was utilized to explore the potential causal linkage between Glaucoma and Anxiety. By conducting this gene-centric analysis, our goal was to surpass the constraints associated with conventional research methodologies, thereby furnishing more robust evidence in favor of a causal connection between Glaucoma and anxiety, as depicted in Figure 1.

## 2 Materials and methods

We conducted a Mendelian Randomization investigation to elucidate the potential causal association between Glaucoma and the susceptibility to anxiety. The MR methodology employs genetic variants as instrumental variables to estimate the causal impact of the exposure (Glaucoma) on the outcome (risk of anxiety), while mitigating the influence of confounding factors. All statistical analyses were executed using the R programming language, employing specialized software packages tailored for MR studies such as TwoSampleMR and Mendelian Randomization.

### 2.1 Data source

We sourced GWAS datasets for Glaucoma (Pubmed ID: GCST90011766) and anxiety (Pubmed ID: GCST007710) with the largest sample sizes from the Integrative Epidemiology Unit OpenGWAS (IEU OpenGWAS) project website.^1^ Raw data can be accessed via the respective publications on the Pubmed website. Data retrieval occurred on March 21, 2024. Both datasets comprised European populations without gender restrictions. The Glaucoma dataset encompassed 14,219,919 SNPs, while the anxiety dataset comprised 18,485,882 SNPs.

### 2.2 Instrumental variable criteria

Criteria for selecting SNPs as instrumental variables were as follows:

(1) The instrumental variables exhibited high correlation with the exposure, with an F-statistic exceeding 10 indicating substantial correlation (11).
(2) Instrumental variables were not directly associated with the outcome but influenced it solely through the exposure, indicating absence of genetic pleiotropy. A pleiotropy test was conducted, with a result of P ≥ 0.05 signifying no genetic pleiotropy.
(3) Instrumental variables were unrelated to unmeasured confounding factors. Since MR-selected SNPs adhere to the genetic principle of random allele allocation from parents to offspring, their susceptibility to environmental and postnatal factors is minimal. Thus, it was theoretically assumed that instrumental variables remained independent of environmental factors such as socioeconomic and cultural influences (12).

### 2.3 SNP selection

Meaningful SNPs were selected from the GWAS summary data of Glaucoma based on a screening criterion of *P* < 5 × 10^–8^. Each SNP’s independence was ensured by setting a linkage disequilibrium coefficient (r^2^) of 0.001 and a linkage disequilibrium region width of 10,000 kb, thereby mitigating the potential influence of genetic pleiotropy (13). Glaucoma-associated SNPs were then extracted from the anxiety GWAS summary data, with a minimum r^2^ > 0.8 to ensure result accuracy. Missing SNPs were directly excluded. The datasets were integrated, and SNPs directly associated with anxiety (*P* < 5 × 10^–8^) were filtered out.

### 2.4 Causal relationship verification

To verify the causal relationship between Glaucoma exposure and anxiety outcome using SNPs as instrumental variables, we employed four regression models: MR-Egger regression, weighted median estimator (WME), inverse-variance weighted (IVW) random-effects model, and simple model. The IVW method directly calculates causal effect estimates using summary data, without the need for individual-level data. MR-Egger regression fits a linear function by assessing the correlation between each SNP and anxiety (Y) and between each SNP and Glaucoma (X). Sensitivity analysis utilized the leave-one-out method. All analyses were conducted using the TwoSampleMR package (version 0.5.11) in R Studio software (version 4.3.3), with a significance level of α = 0.05.

## 3 Results

### 3.1 SNP information screening results

A total of 14,219,919 SNP information was obtained for Glaucoma. After filtering based on a criterion of *P*-value < 5 × 10^–8^, 4,358 SNPs remained. The file “exposure_GLA.csv” was exported and placed in the TwoSampleMR folder. After renaming the sequence names, SNPs were selected to ensure independence by setting a linkage disequilibrium coefficient (r^2^) of 0.001 and a linkage disequilibrium region width of 10,000 kb, excluding the influence of genetic pleiotropy. This resulted in the removal of 4,297 SNPs, leaving 61 SNP data. At this time, the SNP database of anxiety was imported, and the number of SNPs obtained was 18,485,882. Then, the anxiety data and Glaucoma data which just screened were merged, and 60 SNPs were finally obtained (Table 1). Heterogeneity test was carried out on these 60 SNP data, and three sets of outlier data were found, namely data No. 17, 45, and 49. No significant changes were found when they were removed. According to MR Egger regression model, *p* = 0.56, IVW regression model, *p* = 0.56, both of which are greater than 0.05, suggesting that glaucoma does not cause anxiety disorder.

**TABLE 1:** Summary of the selected SNP information.

Number	SNP	CHR	BP	A1	Beta	SE
1	rs10151220	14	34715465	T	−0.0019	0.0015
2	rs10230941	7	117636111	C	0.0013	0.0015
3	rs10248136	7	39077397	T	−0.0022	0.0015
4	rs10517281	4	54027595	A	9.00E−04	0.0015
5	rs10739689	9	129914147	C	6.00E−04	0.0015
6	rs111439095	13	76254433	A	0.0014	0.0015
7	rs1139795	22	19867771	T	0.0013	0.0016
8	rs11658334	17	58830188	A	6.00E−04	0.0017
9	rs11968883	6	158971411	T	0.0022	0.0015
10	rs12208086	6	36586070	A	−0.0026	0.0015
11	rs12540035	7	116159526	A	−0.004	0.0015
12	rs1336980	9	129377855	C	3.00E−04	0.0015
13	rs1649068	10	60304864	A	0.0036	0.0015
14	rs17125973	14	53415359	A	0.0014	0.0015
15	rs17527016	4	111963719	T	−4.00E−04	0.0015
16	rs1972459	7	83287607	A	−0.0021	0.0015
17	rs2113818	2	12890860	T	−0.0016	0.0015
18	rs2472494	9	107695539	T	−0.0022	0.0015
19	rs2514885	8	108277130	T	0.0014	0.0015
20	rs257336	16	65055840	T	0.0054	0.0015
21	rs2579989	6	51460154	T	0	0.0015
22	rs2627761	2	55933014	T	8.00E−04	0.0016
23	rs2667477	12	84023388	T	0.0022	0.0015
24	rs2735114	6	29910034	A	0.001	0.0015
25	rs2745572	6	1548369	A	0.0018	0.0015
26	rs2790049	1	165743523	A	0.0018	0.0015
27	rs2811688	6	134372150	C	−6.00E−04	0.0015
28	rs31916	5	14814883	A	0.0024	0.0015
29	rs33912345	14	60976537	A	9.00E−04	0.0016
30	rs36039219	7	11704538	A	2.00E−04	0.0015
31	rs3753841	1	103379918	A	0.0031	0.0015
32	rs3825942	15	74219582	A	−0.0026	0.0016
33	rs41283694	10	60156574	A	0.0029	0.0016
34	rs41543317	17	44087500	A	−8.00E−04	0.0015
35	rs4414666	2	66537344	T	0	0.0015
36	rs4577906	7	82955177	C	0.0035	0.0015
37	rs4652964	1	38078300	A	0.0012	0.0015
38	rs4653159	1	36579215	A	0.0022	0.0015
39	rs4819641	22	18353630	C	0.0014	0.0015
40	rs55882252	2	153361700	T	0	0.0015
41	rs56233426	3	186128816	A	−0.0028	0.0015
42	rs58073046	11	120248493	A	−0.0011	0.0015
43	rs581796	11	86355565	T	0.003	0.0015
44	rs6117318	20	6507717	A	−9.00E−04	0.0015
45	rs62283809	3	171820211	T	−0.0015	0.0017
46	rs6475604	9	22052734	T	8.00E−04	0.0015
47	rs6490697	13	22679011	T	−0.0017	0.0016
48	rs6602453	10	10840849	A	−1.00E−04	0.0015
49	rs676015	6	2064648	T	−7.00E−04	0.0015
50	rs6845653	4	7899379	T	0.0032	0.0015
51	rs7137828	12	111932800	T	−0.0064	0.0015
52	rs72482850	1	101117684	A	4.00E−04	0.0015
53	rs7275118	20	18010447	T	−5.00E−04	0.0015
54	rs7284245	22	29613441	T	−4.00E−04	0.0016
55	rs7946009	11	128387422	T	−0.0037	0.0015
56	rs7972874	12	28203245	A	−3.00E−04	0.0015
57	rs935328	15	57538801	A	−0.0022	0.0015
58	rs9494457	6	136474794	A	−4.00E−04	0.0015
59	rs9819278	3	85144350	A	−0.0052	0.0015
60	rs9913911	17	10031183	A	1.00E−04	0.0015

SNP, SNP number; CHR, chromosome number; BP: location, A1: effector allele.

### 3.2 Causal relationship verification

**FIGURE 2:**
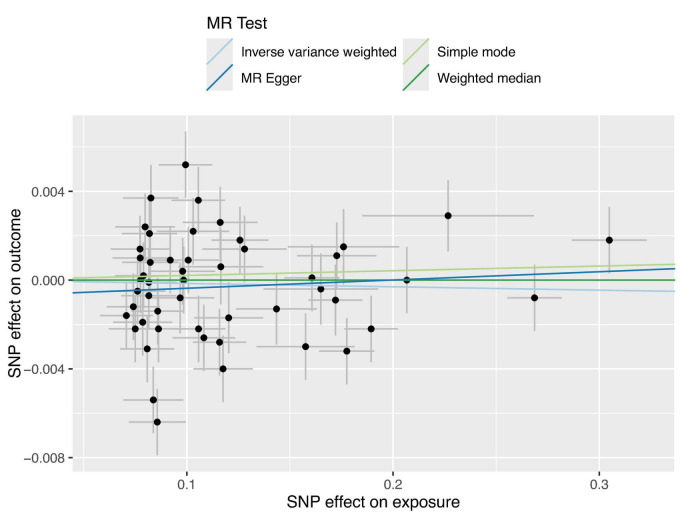
Four Scatter plots of regression models are shown in the figure. Apart from the MR Egger regression line, all other regression lines pass through the origin, and the intercept of the MR Egger regression line with the *y*-axis has an absolute value of less than 0.001. This indicates that there is virtually no apparent confounding in this study.

The regression results of the four methods are shown in Table 2. And all the calculation result of the regression models are greater than 0.05. So this Mendelian randomization study tells us that glaucoma patients do not have a higher incidence of anxiety. The scatter plot is shown in Figure 2.

**TABLE 2:** Regression model results of the four methods.

Four methods MR regression model results				
**Method**	**β**	**se**	**OR (95% CI)**	***P***
MR-Egger	0.003	0.006	1.004(0.991∼1.016)	0.563
WME	0.000	0.003	1.000(0.995∼1.005)	1.000
IVW	0.002	0.003	0.998 (0.993∼1.004)	0.556
Simple mode	0.002	0.006	1.002 (0.992∼1.013)	0.725

WME, weighted median estimator; IVW, inverse-variance weighted.

### 3.3 Sensitivity analysis

**FIGURE 3:**
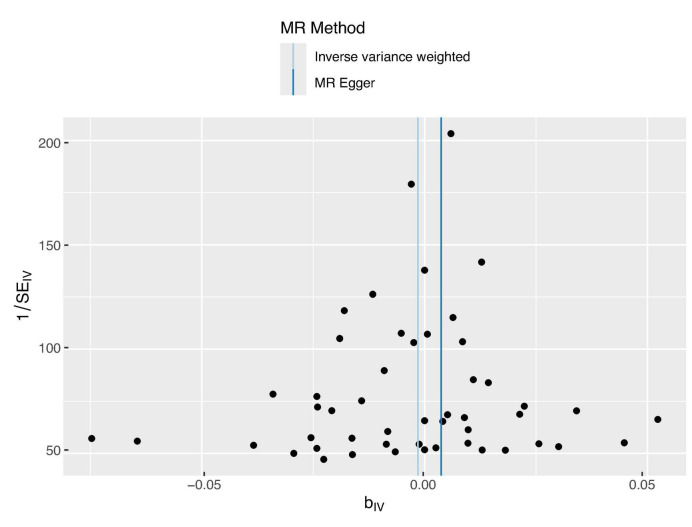
Funnel plot distribution of 44 SNP information. The funnel plot displays good symmetry, suggesting that the SNP variations included in this study are consistent regarding the effect size and direction on the exposure factor, thus indicating low heterogeneity. This supports the reliability of the study results.

**FIGURE 4:**
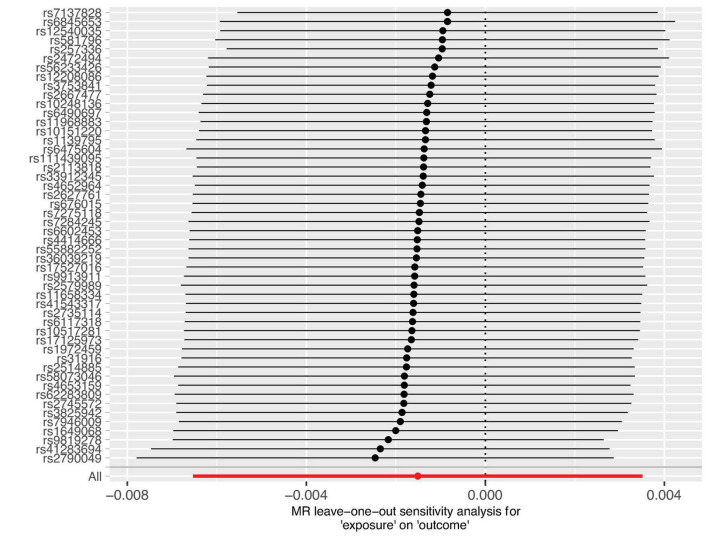
Sensitivity analysis results of the 44 SNP information. All markers observed span the coordinate zero, and excluding any single marker does not alter the conclusions. This demonstrates the robustness of the study findings.

The sensitivity analysis was performed using the leave-one-out method, and the results showed that regardless of which SNP was removed, the conclusions have not changed. This suggests that removing any individual SNP would not have a significant impact on the results, indicating the robustness of the MR findings in this study. The funnel plot and detailed sensitivity analysis results can be found in Figures 3, 4, respectively.

## 4 Discussion

Glaucoma is characterized by optic neuropathy with a hallmark of progressive loss of retinal ganglion cells (14). Currently, there are no effective treatments for the degeneration of these cells, and the primary goal of glaucoma management is to reduce the intraocular pressure (15) and prevent progression (16). Particularly since intraocular pressure is the only treatable risk factor (17) in clinical practice, both doctors and patients give it special attention, making it a chronic condition that requires lifelong care (18). However, chronic illnesses are often associated with psychological disorders such as anxiety (19, 20). Agorastos et al. (21) found that among glaucoma patients, the prevalence of anxiety in those with visual field defects was 44.8%, compared to 24.3% in those without visual field defects (21). However, DY Shin et al. found that patients with anxiety showed faster rates of Retinal Nerve Fiber Layer (RNFL) decline, as measured by Optical Coherence Tomography (OCT) (10).

There is a substantial body of research indicating high prevalence rates of anxiety among glaucoma patients (22, 23). These studies suggest that the heightened incidence of anxiety may stem from the diagnosis of glaucoma itself, driven by concerns over potential blindness, the financial burdens of treatment, and impaired daily activities (24, 25). Anxiety, as stress responses, are thought to originate in the amygdala (26), eliciting neurotransmitter release and stimulating the autonomic nervous system (ANS), which impacts multiple organs (27). The ANS’s response to emotional stress may play a significant role in the development or progression of glaucoma (28, 29). Furthermore, excessive retinal oxidative stress in glaucoma, leading to widespread loss of melanopsin-expressing retinal ganglion cells, plays a critical role in non-visual phototransduction, affecting circadian rhythm changes and melatonin production indirectly (30, 31). Additionally, some glaucoma medications may alter patients’ mood (32).

Despite the abundance of studies indicating a link between glaucoma and increased rates of anxiety, contrasting research from various global scholars suggests that glaucoma patients do not exhibit a heightened probability of suffering from these mental health conditions (33–35). All these clinical investigations have yet to definitively establish the causal relationship between glaucoma and anxiety.

Traditional epidemiological studies are hindered by confounding factors and reverse causality, complicating the determination of the true causal relationship between glaucoma and mental health issues. In this context, MR offers a unique approach, utilizing genetic variants as instrumental variables to estimate the causal effect of one factor on another, circumventing the limitations inherent in the aforementioned study designs (36).

Therefore, we decided to further explore the relationship between glaucoma and anxiety disorders using MR studies. Our findings across various models, including MR Egger regression, Inverse Variance Weighted (IVW) regression, and Weighted Median regression models, indicate that glaucoma does not cause anxiety disorders, with *p*-values of 0.56 for both MR Egger and IVW models, exceeding the threshold of 0.05. Even after excluding three outliers, the conclusion remained unchanged, and sensitivity analyses confirmed the stability of this conclusion. Pleiotropy analysis yielded a *p*-value of 0.38, suggesting that the trial results are reliable and not overly influenced by confounding factors. Additionally, the intercept of the MR-Egger regression line with the *y*-axis being less than 0.001 in Figure 2 also indicates a low likelihood of confounding factors. These MR study results suggest that there is no direct causal link between glaucoma and anxiety, and there are no significant confounding factors at the genetic level. It should be noted that even among Asians, studies have shown significant variability in the prevalence of anxiety among patients with glaucoma. Specifically, the prevalence of anxiety in Japanese glaucoma patients is 13.0% (8), while in Chinese patients, it is 22.9% (37). Notably, the prevalence in Singaporean glaucoma patients reaches as high as 64% (38). Scholars have found that these studies differ in terms of research design, sample size, and demographic characteristics (24). These findings contribute to a better understanding of the relationship between glaucoma and anxiety.

The availability of GWAS data for East Asian and African populations is limited. Although we found an East Asian Glaucoma SNP database through the IEU OpenGWAS project website (PubMed ID: GCST005388), a reliable database related to anxiety in the East Asian population was not found. To ensure the accuracy of our experimental results, we ultimately opted to use a European database. This decision also facilitates future comparisons with research conducted by other scholars. Finally, while this study explored the genetic association between glaucoma and anxiety within a European population database, it holds implications for the prevention and treatment of anxiety caused by glaucoma in other populations and nations. However, we must acknowledge that the lack of analysis of other ethnic groups represents a significant limitation of our research.

It is important to note that this study does not completely exclude the relationship between elevated intraocular pressure and anxiety. This indeed presents an interesting direction for research, which could further elucidate the interpretation of our results. We hope that our team can present findings in this area shortly.

## 5 Conclusion

Through rigorous Mendelian Randomization analysis, our findings indicate that glaucoma is not a causative factor for anxiety, with minimal influence from confounding factors in this study. These findings enhance our understanding of the relationship between glaucoma and anxiety.

## Data availability statement

The original contributions presented in this study are included in the article/supplementary material, further inquiries can be directed to the corresponding author.

## Author contributions

BL: Data curation, Formal analysis, Methodology, Software, Writing – original draft, Writing – review and editing. MX: Data curation, Formal analysis, Methodology, Writing – original draft. L-lC: Data curation, Formal analysis, Funding acquisition, Writing – original draft. D-kL: Funding acquisition, Supervision, Writing – review and editing.

## Footnotes

1
https://gwas.mrcieu.ac.uk
